# The Besrour Papers: Seeking evidence for family medicine

**DOI:** 10.4102/phcfm.v9i1.1559

**Published:** 2017-09-20

**Authors:** David Ponka

**Affiliations:** 1 Department of Family Medicine, University of Ottawa, Canada

It has been said that ‘location is a matter of geography, but global is a matter of the heart’.^1^ This is very much the spirit that guided the creation of the Besrour Centre at the Canadian College of Family Physicians in 2015 and that has guided its mission to generate knowledge about family medicine globally. The Centre believes that family medicine lies at the heart of health systems and provides a much-needed intersection between the clinical domain of primary care and the broader aims of the primary health care sector more generally (Box 1).

BOX 1 Defining terms.

- *Primary health care:* the sum of all elements of a health system meant to address basic health needs, including preventive care. The World Health Organization further subdivides primary health care into four main areas that together ensure a strong primary health care system^2^: universal health coverage, policy, leadership and governance, and primary care.
- *Primary care:* a subset of primary health care. It represents ‘first-contact access for each new need; long-term person (not disease-) focused care; comprehensive care for most health needs; and coordinated care when it must be sought elsewhere’.^3^ It is provided by both physicians (general practitioners and family doctors) and non-physician practitioners.
- *Family medicine:* the subset of primary care provided by family doctors – physicians with additional training in family medicine – and the focus of the Besrour Papers series.

*Source*: Author’s work. Reprinted from Ponka D, Rouleau K, Arya N, et al.^2^

The spread of family medicine is of great interest around the world. This is why the Besrour Centre (http://www.cfpc.ca/The_Besrour_Centre) and Canadian Family Physician (CFP) have been publishing a series of articles on the evolution of the discipline^2,3,4,5^ and why the latest Besrour paper^6^ is an attempt to give a geographic overview of family medicine’s development in each continent. Readers of *The African Journal of Primary Health Care and Family Medicine* will be interested to compare the discipline on the African continent with how it has evolved elsewhere. In addition, Dr John Parks and colleagues at the American Academy of Family Physicians and the Robert Graham Center conducted a comprehensive survey and developed a map of global family medicine in 2013.^7^ While family medicine has been slower to take hold in Africa compared to other parts of the world, we can also show that training there is now well established.

Certainly, surveying the remarkable spread of family medicine is indirect evidence of its utility, but we must do more to synthesise and aggregate data on the discipline. The Besrour Papers series initially deals with the methodological obstacles to studying such a diverse field^2^ (Box 2). The principal obstacle is the lack of a globally accepted definition of what constitutes a ‘family doctor’ because of the diverse roles that we play in our communities. A family doctor in Africa looks very different from a family doctor in Canada and, indeed, family doctors within Africa may look very different from one another. Are there core elements that inextricably bind us together? Some would suggest continuity of care is one such core feature. But this may be less achievable, at least initially, in contexts where clinical demand is overwhelming, such as parts of Africa. Others would point to comprehensiveness of services but, again, these vary greatly according to remoteness and community needs.

BOX 2 Principal methodological obstacles in expanding the evidentiary basis of family medicine globally.

The following are the main methodological challenges:

- Family medicine takes on very different roles (both with patients and in interfacing with the rest of the system) from health system to health system, thus making aggregation of data difficult.
- Most currently available research comes from industrialised contexts.
- In many countries, family medicine competes with other disciplines rather than performing a gatekeeping role.
- Patient continuity (and its benefits) might not be achievable in the short term when clinical demands are great.
- Separating the influence of family physicians from that of other primary care professionals is not always easy.

*Source*: Author’s work. Reprinted from Ponka D, Rouleau K, Arya N, et al.^2^

Perhaps this response to local context and need is a positive core element of our discipline. We should not seek homogeneity just for the sake of study and collating data. But are there any other competencies that we can consider universally fundamental to family medicine? In Canada, we use the Canadian Medical Education Direction for Specialists (CanMEDS) – Family Medicine framework.^8^ The generic CanMEDS framework (medical expert, communicator, collaborator, manager, health advocate, scholar and professional) is adapted with more unique family medicine attributes: being community-based, serving a defined population and having an approach based on the patient–physician relationship. The relationship with patients might not be unique to family doctors, but in the Canadian context, it is central to our role and our identity. Other regions have developed their own frameworks,^5^ and others continue this important process of self-definition. This is an essential prerequisite to continue to generate evidence on family medicine.

Because of these necessary local adaptations and the related diversity, we have also used narratives to better understand common threads in the discipline.^3^ There is perhaps a special role for narrative to describe a heterogeneous field and one based on relationships. And it is very likely that studying family medicine will continue to require a number of mixed-method approaches.

We continue to reach for evidence that family medicine plays, and can play in the future, a central role towards improving global health. Our next strategy, considering the heterogeneity of data that we have described, is to engage in high-quality cross-country comparisons^9^ with our partners in Brazil, another country where family doctors form the backbone of the health system. In doing so, we are reminded of how much we have to learn from one another.
